# Mistreatment of women during childbirth and postpartum depression: secondary analysis of WHO community survey across four countries

**DOI:** 10.1136/bmjgh-2023-011705

**Published:** 2023-08-23

**Authors:** Chris Guure, Philomina Akandity Aviisah, Kwame Adu-Bonsaffoh, Hedieh Mehrtash, Adeniyi Kolade Aderoba, Theresa Azonima Irinyenikan, Mamadou Dioulde Balde, Olusoji Adeyanju, Thae Maung Maung, Özge Tunçalp, Ernest Maya

**Affiliations:** 1 Department of Biostatistics, School of Public Health, University of Ghana, Legon, Ghana; 2 Department of Global Health and Population, Harvard T.H. Chan School of Public Health, Boston, MA, USA; 3 Department of Health Information Management, College of Health and Well-Being, Kintampo, Ghana; 4 Department of Obstetrics Gynaecology, University of Ghana Medical School, University of Ghana, Accra, Ghana; 5 Department of Sexual and Reproductive Health and Research, including UNDP/UNFPA/UNICEF/WHO/World Bank Special Programme of Research, Development and Research Training in Human Reproduction (HRP), World Health Organization, Geneve, Switzerland; 6 Reproductive and Maternal Health, and Healthy Ageing Unit, Universal Health Coverage - Life Course Cluster, World Health Organization Regional Office for Africa, Brazzaville, Democratic Republic of Congo; 7 Department of Obstetrics and Gynaecology, Mother and Child, State Specialist Hospital, Akure, Nigeria; 8 Department of Obstetrics and Gynecology, Faculty of Clinical Sciences, University of Medical Sciences, Akure, Ondo State, Nigeria; 9 Cellulle de Recherche en Sante de la Reproduction en Guinee (CERREGUI), Conakry, Guinea; 10 Adeoyo Maternity Teaching Hospital, Ibadan, Nigeria; 11 Department of Medical Research, Ministry of Health and Sports, Yangon, Myanmar; 12 Department of Population, Family & Reproductive Health, School of Public Health, University of Ghana, Legon-Accra, Ghana

**Keywords:** Mental Health & Psychiatry, Community-based survey, Maternal health

## Abstract

**Background:**

Postpartum depression (PPD) is a leading cause of disability globally with estimated prevalence of approximately 20% in low-income and middle-income countries. This study aims to determine the prevalence and factors associated with PPD following mistreatment during facility-based childbirth.

**Method:**

This secondary analysis used data from the community survey of postpartum women in Ghana, Guinea, Myanmar and Nigeria for the WHO study, ‘How women are treated during facility-based childbirth’. PPD was defined using the Patient Health Questionnaire (PHQ-9) tool. Inferential analyses were done using the generalised ordered partial proportional odds model.

**Results:**

Of the 2672 women, 39.0% (n=1041) developed PPD. 42.2% and 5.2% of mistreated women developed minimal/mild PPD and moderate/severe PPD, respectively. 43.0% and 50.6% of women who experienced verbal abuse and stigma/discrimination, respectively developed minimal/mild PPD. 46.3% of women who experienced physical abuse developed minimal/mild PPD while 7.6% of women who experienced stigma/discrimination developed moderate/severe PPD. In the adjusted model, women who were physically abused, verbally abused and stigma/discrimination compared with those who were not were more likely to experience any form of PPD ((OR: 1.57 (95% CI 1.19 to 2.06)), (OR: 1.42 (95% CI 1.18 to 1.69)) and (OR: 1.69 (95% CI 1.03 to 2.78))), respectively. Being single and having higher education were associated with reduced odds of experiencing PPD.

**Conclusion:**

PPD was significantly prevalent among women who experienced mistreatment during childbirth. Women who were single, and had higher education had lower odds of PPD. Countries should implement women-centred policies and programmes to reduce mistreatment of women and improve women’s postnatal experiences.

**What is already known on this topic**

- Mistreatment of women during facility-based childbirth is a global phenomenon but more common in low-income and middle-income countries.
- Most studies on mistreatment of women during childbirth have reported measures related to physical abuse, verbal abuse, neglect, and stigma and discrimination.
- There is limited evidence using empirical data to explore relationship between mistreatment during facility-based childbirth and postpartum depression (PPD).

**What this study adds**

- This study has shown that PPD is common among postpartum women.
- Women who experience mistreatment during facility-based childbirth have higher odds of experiencing PPD compared with those who did not experience mistreatment.
- Women who were single or have higher education had lower odds of developing PPD.

**How this study might affect research, practice or policy**

- The findings from this study suggests that context-specific policies and programmes need to be implemented in the various countries to reduce mistreatment of women and improve women’s postnatal experiences.
- Pregnant women and postnatal mothers should be supported, educated and empowered to know their health rights to minimise mistreatment during childbirth.
- We recommend implementation of awareness campaigns and early screening, detection and treatment of mothers with PPD.

## Background

Mistreatment of women during facility-based childbirth represents an important cause of infringement on women’s right and dignity.^1 2^ This global public health problem manifests in a variety of forms. These include verbal abuse, physical abuse, stigma and discrimination, poor communication between women and healthcare providers, constraints due to the conditions in the healthcare system, non-consented care, non-confidential care, non-dignified care, abandoning care due to failure to pay, detention at facilities, forced to use unnecessary medication and lack of informed consent for examinations, treatment and invasive procedures.^3–6^

Respectful maternity care is a universal human right for every expectant woman in every setting including postnatal care.^7 8^ Nonetheless, many women experience mistreatment during childbirth in health facilities globally including low/middle-income countries (LMICs).^5 6 9^ A study led by the WHO, reported 42% of 2016 observed women during childbirth and 35% of 2672 women surveyed reported experiencing physical or verbal abuse, stigma or discrimination during childbirth.^6^

Furthermore, mistreatment during childbirth may have both direct and indirect impacts on the health and well-being of the woman and her baby.^8^ Pregnancy and the postpartum period are vulnerable times for maternal mental health; but in low-income nations, many people view symptoms of depression as spiritual or personal issues rather than a medical condition, which requires clinical attention and could be treated.^10^ Depression is a common mental health problem during the postpartum period and is associated with numerous medical and psychosocial problems in both the mother and child.^11^

Depression is a leading cause of disability in women globally with estimated prevalence of approximately 20% in LMICs.^12^ Postpartum depression (PPD) is a vital public health issue.^13^ Findings from a systematic qualitative review exploring what women want from postnatal care found that some women experience periods of low mood, depression and loneliness during the postnatal period.^14^

Causes of PPD are multifactorial. Negative birth experiences including feeling of abandonment during childbirth, are linked to the occurrence of mental health disorders, including PPD and post-traumatic stress disorder.^15^ Studies exploring PPD in Brazil and Russia also reported that mistreatment of women (or disrespect and abuse or negative birth experiences) increase the odds of PPD.^15 16^ Birth experiences such as lack of social support, obstetric difficulties and traumatic childbirth also play a major role in the development of PPD.^16^ Smorti and colleagues in 2019, identified high level of PPD among women who experienced induced labour.^17^ Weobong *et al* also identified peripartum/postpartum complications, newborn ill-health, stillbirth or neonatal death as determinants of postnatal depression in rural Ghana.^18^

In recent years, there have been important advances in documenting the burden of mistreatment of women during maternity care. While there has been an increase in the volume of research reporting prevalence and determinants of mistreatment of women in LMICs, few studies have assessed the consequences of poor care during childbirth on the mental health of women and their newborns in the postnatal period.^15^ This study aims to determine the prevalence and factors associated with PPD following mistreatment during childbirth in some selected public health facilities across four countries.

## Methods

### Data collection and recruitment procedure

This secondary analysis is based on the WHO multi-country study: ‘How women are treated during facility-based childbirth study’.^6^ The details of the data collection methods and tools have been published elsewhere.^6 19^ In brief, consenting women who were at least 15 years old and had childbirth in 12 public health facilities in Ghana, Guinea, Myanmar and Nigeria were recruited. Women were observed during labour and birth. After discharge, the women were followed up and interviewed between 4 and 8 weeks during community surveys at locations agreeable to them outside the health facility. Each study participant had unique identity number that was used for both the labour observation at the facility and the community survey. The data were collected using digital, tablet-based tools (BLU Studio XL2, Android, BLU Products, Miami, Florida, USA) and uploaded to a central monitoring unit (Openclinica server) at WHO in Geneva.

### Study design and participants

The study participants for this analysis were women who took part in the WHO community survey across the four countries.

Twelve maternity units, in general hospitals (three in each country) were involved in the study. All the hospitals were purposively selected. They were included in the study because they were not included in the formative phase of the study, were at least secondary-level facility, conducted at least 200 deliveries per month, had a well-defined catchment area and were willing to allow non-clinicians to observe women in labour. Data collection started first in Nigeria from 19 September 2016 to 26 February 2017, followed by Ghana from 1 August 2017 to 18 January 2018, in Guinea from 1 July to 30 October 2017 and finally in Myanmar from 26 June to 5 September 2017.

The community-based surveys were conducted with the women within 4–8 weeks post partum. Women were eligible for the community survey if they were admitted for childbirth, were at least 15 years old, were willing and able to participate, lived in the catchment area and provided consent. Women were excluded if they were not admitted for childbirth, had first-degree relative in the hospital, were a staff of the hospital, were distressed or were unable to provide consent. Also excluded were women who lived outside the catchment area or could not provide sufficient contact information. We did not seek consent from providers. We have included an author reflexivity statement detailing how we have leverage on our international collaboration to enhance equitable partnership as online supplemental appendix 1.

10.1136/bmjgh-2023-011705.supp1 Supplementary data

### Postpartum instrument

The dependent variable for this study is PPD: this was defined based on the Patient Health Questionnaire (PHQ-9) grade line that was adapted and used.^20^ PHQ-9 tool is effective for detecting and monitoring the severity of depression.^21^ PHQ-9 is a nine-item questionnaire designed to screen for depression in primary care and other medical settings.^20^ This depression module scores each of the nine Diagnostic and Statistical Manual of Mental Disorders, fourth edition (DSM-IV) criteria as ‘0’ (not at all), ‘1’ (several days), ‘2’’ (more than half the days) and ‘3’ (nearly every day), as answers to these nine items. The individual scores are added to arrive at the total final score which is used to establish depressive disorder diagnoses as well as grade depressive symptom severity. The scale has the interpretation of the scores as ‘1–4’ (minimal depression), ‘5–9’ (mild depression), ‘10–14’ (moderate depression), ‘15–19’ (moderately severe depression) and ‘20–27’ (severe depression).^20^ In this analysis, the responses are re-classified into three levels: no depression, mild/minimal (mild and minimal) and moderate/severe (moderate, moderately severe and severe).

### Exposure

There are three primary exposure variables of interest highlighted in previous research and which are based on respondents report of having experienced the following mistreatment items: physical abuse, verbal abuse and stigma and/or discrimination which were reported across all four study countries. Responses were classified as having been mistreated and categorised as dichotomous (yes or no) and analysed as separate exposure variables against the outcome variable.

Physical abuse included pinching, kicking, slapping, punching, gagging, tying, holding down and fundal pressure. Verbal abuse consisted of shouting, scolding, mocking, negative comments about baby’s physical appearance, negative comments on woman’s sexual activity, threatened that mother and baby would have poor outcome and staff hissing at the woman. Stigma and/or discrimination included negative comments regarding woman’s race, religion, age, marital status, educational level and low economic status.

### Statistical analysis

Descriptive and inferential analyses were carried out using complete cases. Initial analyses were done to identify the proportion of women who experienced mistreatment as was reported by the respondents during the follow-up survey. Proportions were obtained for all the categorical variables of interest. For the inferential statistics, we employed the generalised ordered partial proportional odds model due to the ordered number of categories (three) of the predicted variable.

This model is used to quantify the ORs of the predictor on the outcome of interest controlling for identified confounding variables (marital status, maternal age, education, number of pregnancies, number of births, country, mode of delivery and consent for CS in the dataset. The model is specified as follows:

$P\left(Y_{i}>j\right)=g\left(X\beta_{j}\right)=\frac{\exp\left(\alpha_{j}+X_{i}\beta_{j}\right)}{1+\left\{\exp\left(\alpha_{j}+X_{i}\beta_{j}\right)\right\}},\;j=1,2,\ldots ,M-1$(1)

where M represents the number of categories of the ordinal variable, in this case three, that is, minimal, moderate and severe. It can be deduced from equation (1) that the probability that Y will take on each value say, 1,…,M are equal to

$P\left(Y_{i}=1\right)=1-g\left(X_{i}\beta_{1}\right)$(2)

$P\left(Y_{i}=j\right)=g\left(X_{i}\beta_{j-1}\right)-g\left(X_{i}\beta_{j}\right),\;j=2,\ldots ,M-1$(3)

$P\left(Y_{i}=M\right)=g\left(X_{i}\beta_{M-1}\right)$(4)

Based on these expressions, if *M*=2, the generalised ordered partial proportional odds model is equivalent to the standard logistic regression model.

Equation (1) is similar to a parallel-lines model except that in the parallel-lines (proportional odds) model all β’s are constrained to be the same for all values of *j* thereby being equivalent to

$P\left(Y_{i}>j\right)=g\left(X\beta \right)=\frac{\exp\left(\alpha_{j}+X_{i}\beta \right)}{1+\left\{\exp\left(\alpha_{j}+X_{i}\beta \right)\right\}},\;j=1,2,\ldots ,M-1$(5)

The key problem with the parallel-lines model which is mostly used is because of its restrictive assumption on the *β*’s which are likely to be violated due to differences across the values of *j*’s. These restrictive assumptions are easily overcome with the use of the proposed generalised ordered partial proportional odds model.^22^

Variables included in the adjusted model were those that showed significance at p<0.2 (the bivariate level). This model is used to find the statistical association between PPD (no depression, mild/minimal, moderately/severe) and the exposures (physical abuse, verbal abuse, and stigma and/or discrimination) while controlling for confounders. Predictive probabilities were obtained for the three predictors to determine their significance and predictive abilities of the outcome variable of interest (PPD). All analyses were carried out with Stata V.17.

#### Assessment of the parallel regression assumption

A postestimation approach was adopted using the *oparallel* package in Stata to determine whether the parallel regression assumption of an ordered logit model was violated or not. The test specified were the score, Wolfe-Gould and Brant. In all, the global test statistic for each with their corresponding p values were obtained. All were statistically significant indicating a clear violation of the parallel regression assumptions. Therefore, the generalised ordered partial proportional odds model was specified and used for the analyses.

### Patient and public involvement

The design of this study involving four LMICs was the result of a technical consultation meeting that was held at the headquarters of the WHO in November 2013. Among the participants were representatives from other United Nations agencies, academia, research institutions, non-governmental organisations and advocacy groups. Patients were not directly involved in the design of the study.

## Results

### Sociodemographic characteristics of study participants

There was a total of 2672 women included in this analysis. Majority (73.9%) of the respondents were predominantly within the age of 20–34 years. Most (91.3%) of the women were either married or cohabiting and most (81.9%) had vaginal birth.

The overall proportion of the study participants who developed PPD was 39.0% (n=1041). About 35.5% (n=949) developed minimal/mild PPD while 3.4% (92) developed moderate/severe PPD. Single mothers suffered PPD most with 111 cases of depression out of 233 (47.6%). Of the 2672 mothers, 51.2% were either illiterates or had primary education with about 36.9% having developed PPD. Mothers who had attained tertiary education status recorded the highest percentage of PPD cases (44.4%). Majority of the mothers who suffered PPD were mothers that had more than three births (40.1%). PPD was higher among mothers who had vaginal birth (39.4%). By comparison majority (48.5%) of mothers that were sampled(n=561) in Nigeria suffered PPD, followed by Guinea (41.8%, n=645). Mothers from Myanmar recorded the least (26.3%) proportion of PPD (table 1).

**Table 1:** Sociodemographic, and obstetric characteristics and mistreatment during childbirth among postpartum women who had facility-based childbirth

Variables	Total participants	PPD (Yes)	No PPD	Postpartum depression		χ^2^ (p value)
				Minimal/mild	Moderate/severe	
	N=2672	n=1041 (39.0)	n=1631 (61.0)	n=949 (35.5)	n=92 (3.4)	
Maternal age						0.258
≤19	287 (10.7)	112 (39.0)	175 (61.0)	95 (33.1)	17 (5.9)	
20–34	1977 (73.9)	769 (38.9)	1208 (61.2)	703 (35.5)	66 (3.3)	
≥35	408 (15.3)	160 (39.2)	248 (60.8)	151 (37.5)	9 (2.2)	
Marital status						0.016
Single	233 (8.7)	111 (47.6)	122 (52.4)	100 (42.9)	11 (4.7)	
Married/cohabitating	2439 (91.3)	930 (39.1)	1509 (61.9)	849 (34.8)	81 (3.3)	
Education						0.002
No/primary education	1368 (51.2)	506 (36.9)	862 (63.0)	449 (32.8)	57 (4.2)	
Secondary/vocational	775 (29.0)	300 (38.7)	475 (61.3)	284 (36.7)	16 (2.1)	
Tertiary education	529 (19.8)	235 (44.4)	294 (55.6)	216 (40.8)	19 (3.6)	
Number of pregnancies						0.839
1	921 (34.5)	348 (37.8)	573 (62.2)	317 (34.4)	31 (3.4)	
2	649 (24.3)	261 (40.2)	388 (59.8)	244 (37.6)	17 (2.6)	
3	443 (16.6)	172 (38.8)	271 (61.2)	155 (34.9)	17 (3.8)	
≥4	653 (24.4)	258 (39.5)	395 (60.5)	231 (35.4)	27 (4.1)	
None/unknown	6 (0.2)	2 (33.3)	4 (66.7)	2 (33.3)	0 (0.0)	
Number of births						0.825
1	1560 (58.4)	610 (39.1)	950 (60.9)	561 (35.9)	49 (3.1)	
2	511 (19.1)	202 (39.5)	309 (60.5)	184 (36.0)	18 (3.5)	
3	278 (10.4)	99 (35.6)	179 (64.4)	89 (32.0)	10 (3.6)	
≥4	317 (11.9)	127 (40.1)	190 (59.9)	112 (35.3)	15 (4.7)	
Unknown	6 (0.2)	3 (50.0)	3 (50.0)	3 (50.0)	0 (0.0)	
Caesarean section						0.055
Consented	418 (15.6)	149 (35.7)	269 (64.4)	139 (33.3)	10 (2.4)	
Not consented	64 (2.4)	21 (32.8)	43 (67.2)	16 (25.0)	5 (7.8)	
Refuse/don’t	2190 (81.9)	871 (39.8)	1319 (60.2)	794 (36.3)	77 (3.5)	
Mode of delivery						0.850
Vaginal	2187 (81.9)	862 (39.4)	1325 (60.6)	785 (35.9)	77 (3.5)	
Caesarean section	483 (18.1)	178 (36.9)	305 (63.2)	163 (33.8)	15 (16.3)	
Unknown	2 (0.1)	1 (50.0)	1 (50.0)	1 (50.0)	0 (0.0)	
Country						0.000
GHA	836 (31.3)	334 (39.9)	502 (60.1)	314 (37.6)	20 (2.4)	
GUI	644 (24.1)	269 (41.8)	375 (58.2)	214 (33.2)	55 (8.5)	
MMR	631 (23.6)	166 (26.3)	465 (73.7)	159 (25.2)	7 (1.1)	
NGA	561 (21.0)	272 (48.5)	289 (51.5)	262 (46.7)	10 (1.8)	
Mistreatment (abuse)						
Physical abuse						
No	2385 (89.3)		1493 (62.6)	816(34.2)	76 (3.2)	
Yes	287 (10.7)	149 (51.9)	138 (48.1)	133 (46.3)	16 (5.6)	0.000
Verbal abuse						
No	1851 (69.3)		1205 (65.1)	596 (32.2)	50 (2.7)	
Yes	821 (30.7)	395 (48.1)	426 (51.9)	353 (43.0)	42 (5.1)	0.000
Stigma and discrimination						
No	2593 (97.0)		1598 (61.6)	909 (35.1)	86 (3.3)	
Yes	79 (2.9)	46 (58.2)	33 (41.8)	40 (50.6)	6 (7.6)	0.001
Overall						
No	1727 (64.6)		1134 (65.7)	550 (31.9)	43 (2.5)	0.000
Yes	945 (35.4)	448 (47.4)	497 (52.6)	399 (42.2)	49 (5.2)	

Total depression score 0 grouped as no depression, total depression score 1–9 grouped as minimal/mild, total depression score 10–27 grouped as moderate/severe.

GHA, Ghana; GUI, Guinea; MMR, Myanmar; NGA, Nigeria; PPD, postpartum depression.

There were 287 (10.7%) physically abused, 821 (30.7%) verbally abused and 79 (2.9%) experienced stigma or discrimination. Approximately, 35.4% of the total study participants experienced some form of mistreatment during facility-based childbirth. Majority (50.6%) of the mothers who suffered mistreatment developed minimal/mild PPD. Mothers who suffered severe PPD most were those who experienced stigma (7.6%). Of the total women who experience physical abuse (n=149), 51.9% developed PPD, out of this proportion 46.3% developed minimal/mild PPD. Regarding verbal abuse, 5.1% of women who experience it developed moderate/severe PPD. In all 47.4% of women who experienced any form of mistreatment during childbirth developed PPD (n=448) (table 1).

### Proportional odds for no against mild/minimal and moderate/severe PPD

All the primary exposures (physical abuse, verbal abuse and stigma) as well as the confounding variables were statistically significant in predicting no depression against mild minimal, moderate and severe PPD at an alpha level of 5% except maternal age, number of births, number of pregnancies and mode of delivery. There was a 52% (OR=1.52, CI 1.18 to 1.96), 57% (OR=1.57, CI 1.32 to 1.87) and 80% (OR=1.80, CI 1.12 to 2.89) statistically significant increase in PPD among respondents who were physically and verbally abused and stigmatised for the second adjusted model with only the three primary exposures of interest. This was slightly increased to 57% (OR=1.57, CI 1.19 to 2.06), and reduced to 42% (OR=1.42, CI 1.18 to 1.69) and 69% (OR=1.69, CI 1.03 to 2.78) to have any form of PPD against no depression in the overall model. While mothers with tertiary educational status are 32% less likely of being PPD, single mothers have 36% (OR=0.64, CI 0.47 to 0.87) reduced chance PPD. Participants from Myanmar had a 91% increased (OR=1.91, CI 1.49 to 2.45) odds of having PPD compared with Ghana (table 2).

**Table 2:** Determinants of postpartum depression among women who experience mistreatment during childbirth in health facilities

	Unadjusted OR (95% CI)	Adjusted OR (95% CI)—PE	Adjusted OR (95% CI)
No depression vs depression (mild, minimal, moderate and severe)			
Physical abuse			
Yes	1.81 (1.41 to 2.31)***	1.52 (1.18 to 1.96)**	1.57 (1.19 to 2.06)**
No	Ref	Ref	Ref
Verbal abuse			
Yes	1.72 (1.46 to 2.04)***	1.57 (1.32 to 1.87)***	1.42 (1.18 to 1.69)***
No	Ref	Ref	Ref
Stigma and discrimination			
Yes	2.24 (1.42 to3.53)**	1.80 (1.12 to 2.89)*	1.69 (1.03 to 2.78)*
No	Ref	Ref	Ref
Maternal age (years)			
≤19	1.01 (0.74 to 1.37)	–	1.29 (0.85 to 1.97)
20–24	1.00 (0.77 to 1.30)	–	1.13 (0.81 to 1.56)
25–29	1.06 (0.83 to1.35)	–	1.26 (0.95 to 1.67)
30–34	0.97 (0.76 to1.25)		1.15 (0.88 to 1.51)
35+	Ref		Ref
Marital status			
Single	0.68 (0.52 to 0.89)**	–	0.64 (0.47 to 0.87)**
Married/cohabitating	Ref		Ref
Education			
No/primary education	Ref		Ref
Secondary education (Ref)	0.93 (0.78 to 1.11)	–	1.02 (0.83 to 1.25)
Tertiary education	0.73 (0.59 to 0.90)**	–	0.68 (0.53 to 0.88)**
Country			
GHA	Ref		Ref
GUI	0.93 (0.75 to 1.14)	–	0.83 (0.65 to 1.06)
MMR	1.86 (1.45 to 2.33)***	–	1.91 (1.49 to 2.45)***
NGA	0.71 (0.7 to 0.88)**	–	0.84 (0.66 to 1.08)
Number of pregnancies			
1	Ref		Ref
2	0.90 (0.73 to 1.12)	–	0.95 (0.73 to 1.24)
3	0.96 (0.76 to 1.21)	–	0.85 (0.62 to 1.17)
≥4	0.93 (0.76 to 1.14)	–	0.96 (0.69 to 1.34)
Mode of delivery			
Vaginal	0.89 (0.73 to 1.10)	–	1.22 (0.98 to 1.53)
Caesarean	Ref		Ref
Number of births			
1	Ref		Ref
2	0.98 (0.80 to 1.21)	–	0.99 (0.76 to 1.3)
3	1.16 (0.89 to 1.51)	–	1.26 (0.90 to 1.77)
≥4	0.96 (0.75 to 1.23)	–	0.95 (0.66 to 1.35)

*P value<0.05; **p value<0.01; ***p value<0.001.

GHA, Ghana; GUI, Guinea; MMR, Myanmar; NGA, Nigeria; PE, primary exposure; ref, reference.

### Proportional odds for both no and mild/minimal against moderate/severe PPD

All the primary exposures in the unadjusted model were statistically significant in predicting the combination of no depression and mild/minimal versus moderately/severe PPD at an alpha level of 5% except maternal age, number of births, pregnancies and mode of delivery. For the primary exposure variables, there were 42% (OR=1.42, CI 1.10 to 1.83) and 46% (OR=1.46, CI 1.23 to 1.74) statistically significant increase in the odds of PPD among participants who were physically and verbally abused. This was marginally reduced to 32% (OR=1.32 CI 1.00 to 1.74) and 36% (OR=1.36, CI 1.13 to 1.64) respectively for physical and verbal abuse in the overall adjusted model. There was a 62% (OR=1.62, CI 1.20 to 2.19) statistically significant increase odds of being moderately/severely depressed among participants from Myanmar and a 32% (OR=0.68, CI 0.52 to 0.89) statistically significant decreased chance of moderate/severe PPD among participants from Nigeria when compared with participants from Ghana. Participants who had their babies by spontaneous vaginal delivery were 42% (OR=1.42, CI 1.10 to 1.83) more likely of having moderate/severe PPD (table 3).

**Table 3:** Determinants of moderate/severe postpartum depression among women who experience mistreatment during childbirth in health facilities

	Unadjusted OR (95% CI)	Adjusted OR (95% CI)—PE	Adjusted OR (95% CI)
No depression and mild/minimal versus moderate/severe depression			
Physical abuse			
Yes	1.66 (1.30 to 2.13)***	1.42 (1.10 to 1.83)**	1.32 (1.00 to 1.74)*
No	Ref	Ref	Ref
Verbal			
Yes	1.59 (1.34 to 1.88)***	1.46 (1.23 to 1.74)***	1.36 (1.13 to 1.64)**
No	Ref	Ref	Ref
Stigma and discrimination			
Yes	1.90 (1.21 to 2.98)**	1.55 (0.98 to 2.46)	1.52 (0.94 to 2.47)
No	Ref	Ref	Ref
Maternal age (years)			
≤19	1.19 (0.86 to 1.63)	–	1.54 (0.96 to 2.45)
20–24	1.09 (0.84 to 1.42)	–	1.11 (0.77 to 1.59)
25–29	1.09 (0.85 to 1.39)	–	1.02 (0.73 to 1.42)
30–34	1.02 (0.79 to 1.31)	–	1.05 (0.78 to 1.42)
35+	Ref		Ref
Marital status			
Single	0.71 (0.54 to 0.93)*	–	0.79 (0.57 to 1.09)
Married/cohabitating	Ref		Ref
Education			
No/primary education	Ref		Ref
Secondary education	0.84 (0.70 to 1.02)	–	0.86 (0.69 to 1.08)
Tertiary education	0.71 (0.58 to 0.87)**	–	1.05 (0.79 to 1.39)
Country			
GHA	Ref		Ref
GUI	1.21 (0.97 to 1.49)	–	1.16 (0.89 to 1.51)
MMR	1.79 (1.42 to 2.24)***	–	1.62 (1.20 to 2.19)**
NGA	0.69 (0.55 to 0.85)**	–	0.68 (0.52 to 0.89)**
Number of pregnancies			
1	Ref		Ref
2	0.87 (0.71 to 1.07)	–	0.89 (0.68 to 1.18)
3	0.98 (0.77 to 1.24)	–	1.16 (0.81 to 1.63)
≥4	0.96 (0.78 to 1.18)	–	1.25 (0.87 to 1.79)
Mode of delivery			
Vaginal	0.91 (0.74 to 1.12)	–	1.42 (1.11 to 1.83)**
Caesarean	Ref		Ref
Number of births			
1	Ref		Ref
2	0.99 (0.81 to 1.23)	–	1.02 (0.78 to 1.34)
3	1.19 (0.91 to 1.57)	–	1.08 (0.76 to 1.55)
≥4	1.03 (0.79 to 1.32)	–	1.06 (0.72 to 1.55)

*P value<0.05; **p value<0.01; ***p-value<0.001.

GHA, Ghana; GUI, Guinea; MMR, Myanmar; NGA, Nigeria; PE, primary exposure; ref, reference.

### Predictive probabilities of the three primary exposure variables

**Figure 1:**
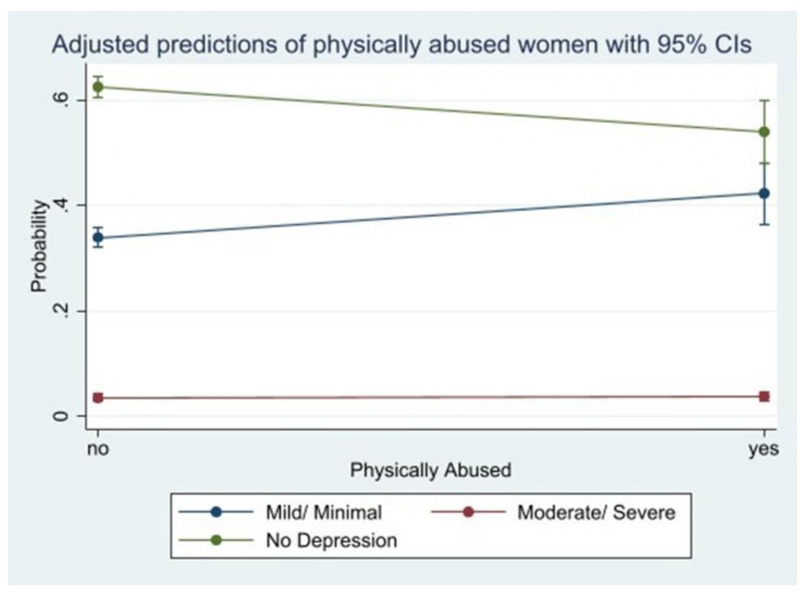
Predictive probability of physical abuse and postpartum depression.

**Figure 2:**
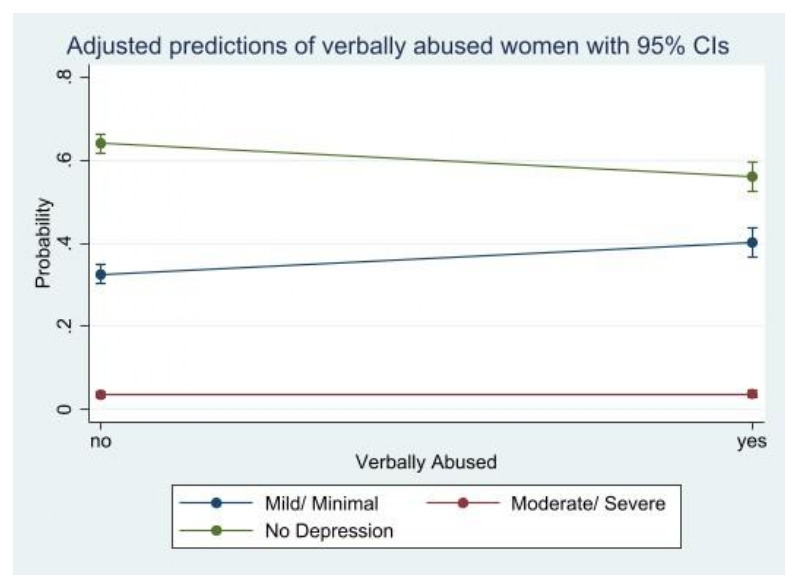
Predictive probability of verbal abuse and postpartum depression.

**Figure 3:**
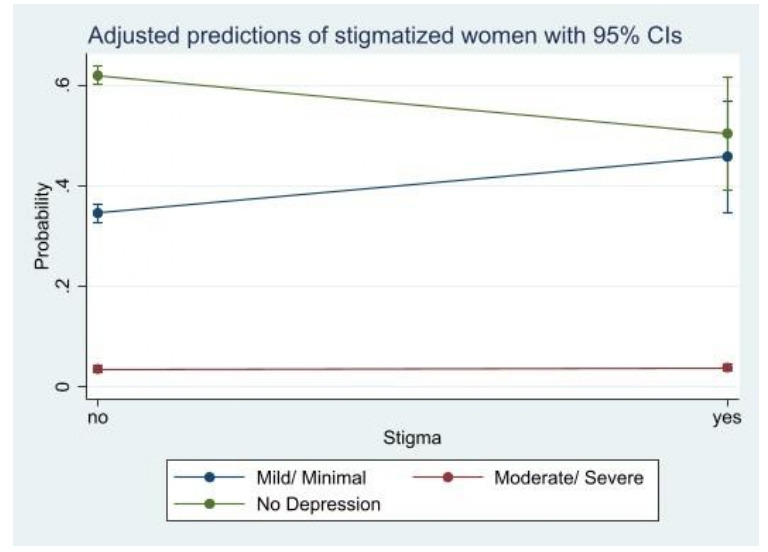
Predictive probability of stigmatisation and postpartum depression.

Women who are not physically and verbally abused and stigmatised have a very high predictive probability (62.5%), (64.0%) and (61.9%), respectively of not having PPD. We observed a moderately high predictive probability among participants who answered yes to being physically abused (54.0%), verbally abused (56.1%) and stigmatised (50.4%) but being classified under no PPD. Being physically (42.2%) and verbally (40.2%) abused and stigmatised (45.7%) have a high predictive probability of mild/minimal PPD. We observed a similar statistically significant predictive probabilities among participants who were moderately/severely depressed with regards to being physically abused (3.8%), verbally abused (3.7%) and stigmatised (3.8%) (figures 1–3).

## Discussion

We report findings on PPD from a community-based survey among 2672 postpartum women who had facility-based childbirth across four countries. In our findings, 47.4% of the women who experienced mistreatment (physical abuse, verbal abuse, and stigma and discrimination) developed PPD. In a recent study conducted in Nigeria, Adeyemo and colleagues reported a high PPD prevalence of nearly 36%.^23^ This could be because women experience significant hormonal changes and fluctuations during and after pregnancy leading to mood changes and instability.^24^ All the primary exposure (physical, verbal, and stigma and discrimination) mistreatment variables included in this analysis as provided in the tables and the predictive (margins plot) analysis, showed significant influence on increasing the odds and probabilities of PPD. This increase is more among postpartum mothers who experience mistreatment during childbirth. This suggests that mistreatment during childbirth increases the risk of PPD occurrence.

There was a positive association between postpartum women who reported that they were physically or verbally abused and the development of moderate/severe PPD relative to those with no PPD and/or those with mild or minimal PPD. Women who were verbally abused during childbirth were almost two times more likely to develop severe symptoms of PPD as was also reported in a survey of the Pelotas birth cohort in 2019.^15^ Similarly, physical abuse of women during childbirth positively influences their chance of developing moderate/severe PPD relative to not developing PPD at all or developing mild/minimal PPD (adjusted OR: 1.57; 95% CI 1.19 to 2.06). This aligns with the findings that abused women are significantly more depressed than the non-abused.^25^ As postulated elsewhere ‘disrespect and abuse towards women during childbirth may contribute to the development of postpartum depression’.^26^ These results affirm their findings that there is a positive association between mistreatment (or institutional violence in obstetric care) and PPD. This may also be a result of women reporting inadequate support due to early discharge and not receiving timely support during childbirth.^14^

In terms of women’s characteristics, our findings show differences in women’s vulnerabilities of self-reporting PPD. Maternal age was not a statistically significant predictor of PPD, contrary to the results of Smorti and colleagues.^17^ The findings of this survey contradicts a study conducted in Nigeria where they found PPD to be common among single than married women.^27^ They attributed their findings to the conjecture that being single and pregnant is associated with economic hardships and stigmatisation, particularly in traditional African settings.^18^ Women who gave birth by spontaneous vaginal birth reported increased odds of PPD. This is contrary to a similar study that intimated that vaginal birth among respondents was observed as natural and normal and so had a less increased risk for PPD.^28^

Assessing the confounders to developing severe PPD, it was realised that attaining tertiary education significantly decrease the odds of developing moderate/severe PPD compared with no PPD and/or having mild or minimal PPD. It is possible that these women by virtue of their higher educational level are gainfully employed and may command some level of respect among healthcare workers. It is stipulated that working mothers however are especially vulnerable to workplace stressors because of sleep deprivation and inability to engage in health promotion activities because of competing demands from home and work.^29^

Women in Myanmar had an increased probability of developing any score of PPD, while women who delivered in Nigeria had a decreased probability of developing PPD when compared with their Ghanaian counterparts. This affirms the fact that the prevalence of PPD may be different between populations and its manifestations may vary across cultures.^30^ These differences imply that a woman’s susceptibility to PPD is based on several factors including social, economic, psychological and biological factors.^31 32^ A postnatal experience where women adjust to their new realities of motherhood and parenting in their own cultural context is essential to achieve positive motherhood.^14^

### Strengths and limitations

The strengths of this study include it being a multi-country study, large sample size and the use of a standardised tool across multiple countries. The paper highlights important findings related to PPD with relevant clinical and research implications. Although the PHQ-9 tool has been validated in Ghana^33^ and Nigeria,^34^ it has not been validated in Guinea and Myanmar. As a result, the estimates of PPD from Guinea and Myanmar may have to be interpreted with caution.

### Implications for future research and practice

While the focus of quantitative measurement studies on mistreatment of women has primarily been conducted during the intrapartum period, qualitative evidence across maternity care has emphasised that women’s experiences of care across pregnancy, childbirth and the postnatal period should be a continuum, and not as three distinct and unrelated states.^35^ Future studies should build on studying the effects of mistreatment during the postnatal period and the health of both mothers and their newborn.

Strategies need to be put in place to educate healthcare providers of the consequences of mistreatment during childbirth. Literature suggests that many forms of disrespect and abuse during childbirth are normalised so they are not considered a problem.^15^ Future research on strategies for prevention of mistreatment during childbirth and control and service support should be implemented to reduce the likelihood of PPD, facilitate the early identification of cases so that they receive adequate treatment and support.

Psychosocial and/or psychological interventions during the antenatal and postnatal period are recommended to prevent PPD and anxiety.^7^ Specialised training and support for healthcare providers is an opportunity to improve women’s experiences of care in the postnatal period and improve future health-seeking behaviour, especially for first-time mothers. Women want to feel ‘cared for’ during the postnatal period as they navigate the transition to motherhood and recovery from childbirth. Postnatal contacts provide an opportunity for healthcare providers to facilitate respectful, individualised, person-centred care at every contact including but not limited to screening for PPD, monitor the baby’s growth and overall health status, treat childbirth-related complications, counsel women about their family planning options and refer the mother and baby for specialised care if necessary.^14^

## Conclusion

Our study has shown that, beyond the abuse of their human rights, women who are mistreated during facility-based childbirth risk experiencing PPD. Women who were single, and those who have higher education had lower odds of PPD. This calls for women to be treated with respect and dignity during facility-based childbirth.

The findings of this study can be used as evidence in the study countries to inform policies and programmes that will ensure that all women have positive pregnancy and childbirth experiences, and improved postnatal experiences supported by empowered healthcare providers within well-functioning health systems. It is essential to reformulate the training of health professionals to promote a more humanised view among facility-based childbirth. Awareness campaigns, screening for early detection and treatment among postpartum mothers is also very necessary to reduce any further impact on mother and child.

## Data Availability

Data are available upon reasonable request.
