# Supplementary material for: Mistreatment of women during childbirth and postpartum depression: secondary analysis of WHO community survey across four countries
Source: BMJ Glob Health. 2023 Aug 23;8(8):e011705. doi: 10.1136/bmjgh-2023-011705 (PMC10450127; doi:10.1136/bmjgh-2023-011705)
Supplement: Supplementary data [file bmjgh-2023-011705supp001.pdf]

Appendix 1 – Reflexivity Statement

1. How does this study address local research and policy priorities?

This research study, which was in two phases was designed as a research collaboration to develop and validate study tools for measuring mistreatment of women during facility-based childbirth. Mistreatment of women during childbirth is a global maternal health problem with grave consequences for both women and their newborns. This is a problem in the study countries. With accurate description and measurement of mistreatment, effective interventions could be put in place to prevent and manage it. This will also enhance monitoring the progress of interventions. Again, with standardized tools, appropriate comparisons can be made within and between countries. This study started with Ghana, Nigeria and Myanmar and was later joined by the team from Guinea (CERREGUI). For this analysis and manuscript, the Ghanaian team (CG, EM, KA-B) came up with the concept note with inputs from our WHO partners (HM and OT) based on the local need for research into maternal mental health which has not been given much attention and its association with mistreatment during childbirth.

2. How were local researchers involved in study design?

The overall study was designed with inputs from the whole study group. At the early stages of the project, a meeting was held at the WHO headquarters, Geneva in 2014. The country research teams (KA-B, EM–Ghana, TMM–Myanmar, MDB–Guinea and Prof Bukola Fawole-Nigeria) were involved in the deliberations to choose appropriate study designs based on their local context together with the WHO research team (HM, OT). Prof Bukola Fawole later passed on to glory before the completion of the study and was duly acknowledged by dedicating the primary paper of the second phase of the study to his memory. CG, KA-B and EM were the Ghanaian study site coordinators and conceptualized this analysis with inputs from HM and OT. The Ghanaian team led the analysis and writing of this manuscript on behalf of the whole research team. PAA joined the research team for this analysis and drafting of the initial manuscript based on her research needs as a PhD student at the University of Ghana, School of Public Health.

3. How has funding been used to support the local research team?

At the start of the study, a research capacity building plan was developed by the local research teams and their WHO counterparts, with support from the HRP Alliance for Research Capacity Strengthening. During the project, the WHO research team led by OT

facilitated a two weeks workshop on data analysis with the research teams from Ghana, Guinea and Nigeria (1-Accra, Ghana 2015, with over 20 members) including KA-B, EM, MDB and others not named on this particular paper, 2- Yangon, Myanmar 2015, involving 14 members of the Myanmar research team and other researchers from the Department of Medical Research) and three week-long workshops on scientific writing (1-Conakry, Guinea 2016, 2-Yangon, Myanmar 2016, 3-Melbourne, Australia 2019). Funding was also used to facilitate dissemination workshops in Guinea, where the team led by MDB shared the results and implications from the study with the Ministry of Health, professional associations, WHO-Guinea and other key stakeholders:https://www.who.int/news/item/15-05-2020-research-leads-to-actions-improving-childbirth-in-guinea. The study also contributed partly to KA-B and TMM's PhD dissertations. TMM's PhD at Khon Kaen University Thailand was sponsored with a scholarship from the HRP Alliance for Research Capacity Strengthening). KA-B's PhD at Utrecht University, The Netherlands, was funded by the Julius Global Health Support Program (Scholarship), University Medical Center Utrecht in the Netherlands.

4. How are research staff who conducted data collection acknowledged?

This paper is one of 18 papers (7 qualitative from the formative phase, 1 protocol, 1 methodological development, and 9 quantitative from the measurement phase) from our research collaboration. Among these 18 papers, research staff who collected data from all 4 study countries have contributed as co-authors on at least 1 paper. Each country's research team has led at least 4 papers with their teams. In all the papers, research assistants who collected data have been duly acknowledged.

5. Do all members of the research partnership have access to study data?

All members of this research partnership have full access to the data. This is evidenced by the number of published journal articles that have come out from this study with lead authors from all the partner countries including this paper.

6. How was data used to develop analytical skills within the partnership?

The research team has worked together in the data analysis throughout the whole study and the current paper. The data analysis workshops and the scientific writing workshops that took place as part of the research partnership helped to strengthen the analytic and writing skills for the research team. For this paper, the Ghanaian team (CG, PAA, KA-B and EM) led the analysis with support from HM and OT.

7. How have research partners collaborated in interpreting study data?

Throughout the research, all partners have been involved in interpreting the study data during data analysis. In particular, for multi-country papers emanating from our research partnership, discussions are held to understand the issues and develop the implications for each country's research, policy and practice.

8. **How were research partners supported to develop writing skills?**

The research team writing this paper is made up of all levels (senior, mid, junior) academics and clinicians. KA-B, PAA, AA and HM are currently working on their PhDs, and they are supported by OT and CG. The authors were supported by the WHO research team (HM OT) to develop and refine their writing skills through regular reviewing of the manuscript and providing constructive feedback. The scientific writing workshops also contributed towards developing the writing skills of the researchers.

9. How will research products be shared to address local needs?

All papers arising from this research partnership have been published in open access journals. After the publication too, plans were developed for the dissemination of our findings within WHO and our individual institutions. Significantly, the standardized tools for measuring mistreatment during facility-based childbirth are also freely available to be used by other researchers.

10. How is the leadership, contribution and ownership of this work by LMIC researchers recognised within the authorship?

To ensure fairness in the leadership, contribution, and ownership of the work, a clear data use and authorship guidance were drawn and discussed with research teams from the study countries and WHO. Among the Ghanaian authors who led this work CG, PAA and KA-B are the first, second and third authors respectively. EM is the last and also the corresponding author. In addition, 9 out of the 11 authors are researchers from the study countries (Ghana, Guinea, Myanmar, Nigeria).

11. How have early career researchers across the partnership been included within the authorship team?

The study teams include early career researchers and clinician researchers (CG, PAA, KAB, HM, OA, TAI, TMM, EM) within the authorship team. They contributed to the data collection, analysis plan, analysis, and writing. It is worth mentioning that, only 1 out of the 8 early career researchers is based in a high-income country. The remaining are based in low-income countries (Ghana, Guinea, Myanmar, Nigeria).

12. How has gender balance been addressed within the authorship?

Gender balance has always been considered within the authorship. Four of the authors are females (PAA, HM, TAI, OT) and seven are males (CG, KA-B, AA, MDB, OA,TMM,EM).

13. How has the project contributed to training of LMIC researchers?

The overall study has also contributed to KA-B and TMM's PhD dissertations. TMM's PhD was made possible through a scholarship from the HRP Alliance for Research Capacity Strengthening). PAA, a PhD student, used the analysis and the initial drafting of the manuscript to improve on her research skills. The data analysis and writing workshops which were carried out as part of this research project also contributed to addressing the research training needs of the study countries.

14. How has the project contributed to improvements in local infrastructure?

This project has not directly contributed to improvements in local infrastructure. However, tablets that were procured for the study countries, have been used for other studies after this collaborative research.

15. What safeguarding procedures were used to protect local study participants and researchers?

The current study was adapted based on local context to safeguard the safety of our research team and study participants. For instance, in Myanmar labour observations were not made because it was considered inappropriate or unsafe to observe labour in the wards of public hospitals. We used all female data collectors due to the sensitive nature of the study. Again, the research team had a study site obstetrician and a senior midwife who were at hand to handle any issues arising. For instance, if a research assistant observes that a woman in labour (including those who are not study participants) is being abuse excessively, she will inform the obstetrician or senior midwife for immediate redress. Finally, there was regular debriefing and reflexive discussions between data collectors, country study teams and our partners from WHO which helped to address any teething issues during data

collection. This process of reflection during data collection is very important to any research involving sensitive topics or violence to ensure the safety of both study participants and research teams.
